# Discussion table on computer science

**DOI:** 10.1093/eurpub/ckac129.232

**Published:** 2022-10-25

**Authors:** D Diethei

**Affiliations:** 1 Human-Computer Interaction, University of Bremen, Bremen, Germany; 2 Leibniz ScienceCampus Digital Public Health, Bremen, Germany

## Abstract

Daniel Diethei is an expert in computer science, human-computer interaction and mobile health. He will support the workshop participants during the world coffee at the discussion table for computer science.

